# The relationship between sedentary behavior and non-suicidal self-injury behavior among adolescents in China

**DOI:** 10.3389/fpsyt.2024.1489707

**Published:** 2024-12-04

**Authors:** Yaru Guo, Xiaojian Yin, Jianyi Xu, Fule Chen, Feng Zhang, Yuan Liu, Pengwei Sun, Jun Hong, Yanyan Hu

**Affiliations:** ^1^ Key Laboratory of Adolescent Health Assessment and Exercise Intervention, Ministry of Education, College of Physical Education & Health, East China Normal University, Shanghai, China; ^2^ College of Economics and Management, Shanghai Institute of Technology, Shanghai, China; ^3^ Physical Education Department, Xiamen Shuangshi Middle School of Fujian, Xiamen, China; ^4^ Physical Education Department, Guangxi Vocational Normal University, Guangxi, China; ^5^ Physical Education College, Shanghai University, Shanghai, China

**Keywords:** adolescents, non-suicidal self-injury, sedentary behavior, screen time, mental health

## Abstract

**Background:**

The objective of this study was to explore the relationship between sedentary behavior and non-suicidal self-injury (NSSI), and to provide a theoretical basis for preventing and reducing the occurrence of NSSI behavior and the development of intervention measures.

**Methods:**

Between September and December 2021, a sedentary behavior and NSSI survey was administered to 10327 Chinese adolescents aged 12–17 who had been randomly selected using the stratified cluster random sampling method. The results were analyzed using the chi-square test and logistic regression analysis to explore the relationship between sedentary behavior and NSSI in this population.

**Results:**

The study found an overall NSSI detection rate among the participants of 25.1%, with occasional NSSI and frequent NSSI detection rates of 11.0% and 14.1%, respectively. The NSSI detection rate is higher in girls than in boys. After controlling for related influencing factors, the NSSI detection rates among adolescents with sedentary behavior duration ≥8 hours and screen time ≥2 hours were 1.393(*β*= 0.332, *P* <0.01) and 1.569(*β*= 0.451, *P* <0.01) times higher than those with sedentary behavior duration <8 hours and screen time <2 hours, respectively.

**Conclusion:**

Sedentary behavior, especially that related to screen time is closely related to the occurrence of NSSI in adolescents. To reduce the occurrence of NSSI, we should therefore pay attention to the harmful effects of sedentary behavior on the physical and mental health of teenagers, take corresponding measures to limit sedentary behavior and screen time, and guide teenagers to use electronic devices correctly.

## 1 Introduction

Non-suicidal self-injury (NSSI) is a significant predictor of suicidal ideation and behavior among adolescents. With the increasing frequency and severity of adolescent NSSI behavior, the risk of suicidal ideation and attempted suicide will increase (1). Adolescent NSSI behavior has become a public health problems that seriously endanger the physical and mental health of teenagers. In China, the social and cultural factors such as academic pressure, the one-child policy's legacy, and the rapid societal changes contribute to the unique context in which NSSI behaviors may emerge and persist. The severity of NSSI is underscored by its association with long-term mental health issues and the need for immediate intervention.

Studies have reported that the global detection rate of NSSI behavior among adolescents is 17.2% (2), and the incidence of NSSI behavior among adolescents in China is also concerning, with a detection rate that ranges from 11% to 23%, and shows a significant growth trend (3). The high incidence, recurrence, and serious clinical risks of NSSI behavior have prompted many psychologists and neurologists to explore its related influencing factors and occurrence mechanisms. Research has found that NSSI behavior may be caused by the combined effects of various influencing factors, including demographic and sociological, psychological, behavioral, and neurobiological factors (4–6). However, different studies report significant differences in influencing factors, and the intervention programs that target the known risk factors generally have modest effects. There is therefore a need for studies to explore other risk factors for NSSI behavior to generate new ideas and serve as references for its prevention and intervention.

The occurrence and development of chronic diseases such as obesity, cardiovascular disease, bone loss, and psychological diseases such as depression, anxiety, and suicidal behavior in adolescents have been associated with sedentary behavior and screen time (7–9). Sedentary behavior patterns undergo significant changes during adolescence and have a serious and negative impact on the physical and mental health of teenagers, but the relationship between sedentary behavior and adolescent NSSI behavior has not yet received sufficient domestic and international research attention. Relevant investigations report that the global average daily sedentary behavior duration of children and adolescents is more than eight hours (10–12). Studies have confirmed that even if the level of physical activity reaches the recommended amount, too much time spent sitting will increase all-cause mortality, and the health risks of sedentary behavior are independent of physical activity (13). The high incidences of excessive sedentary behavior and NSSI among adolescents represent two major public health problems worldwide. At present, the domestic research on the NSSI behavior of adolescents is focused on its epidemiology. Additionally, existing research abroad has only investigated the relationship between NSSI behavior and sedentary behavior involving screen time. However, an independent relationship between the two has not yet been confirmed. Research on the impact of sedentary behavior on NSSI behavior is even more scarce.

This study intends to contribute to this gap in our knowledge by exploring the dose–effect relationship between sedentary behavior and NSSI behavior and identifying the influence of sedentary behavior—especially screen time—on NSSI behavior. From the perspective of physical activity, it provides a relevant theoretical basis for preventing, reducing, and intervening with in the occurrence of NSSI among adolescents as well as further improving the physical and mental health of teenagers in China. By focusing on Chinese adolescents, this study addresses a significant and growing public health concern within a population that represents a substantial proportion of the global youth demographic.

## 2 Materials and methods

### 2.1 Study design and data source

The sample size for this study was determined using the epidemiological survey formula: N=deff u^2^p(1–p)/δ^2^, which comprehensively considered the design effect (deff = 0.8), the Z-score corresponding to a significance level of α=0.05 (u = 1.96), the expected detection rate (p = 20%), and the allowable error (δ = 0.10p) (2, 14, 15). According to this calculation, each age group required 1,229 individuals, totaling 7,374 individuals across six age groups. To compensate for an expected non-response rate of 10%, the final sample size was determined to be 8,193 individuals. After excluding invalid questionnaires and missing data, a total of 10,327 participants were recruited, with a recovery rate of 95.41%, meeting the sample size requirements.

**Figure 1:**
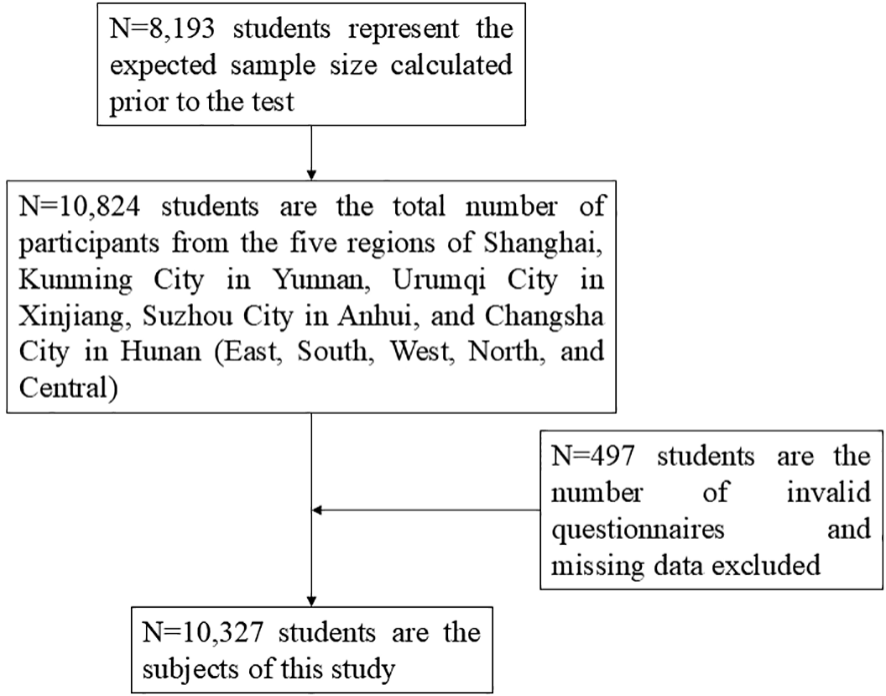
Flowchart of participant involvement.

From September to December 2021, we conducted a field survey and test in five cities in China: Shanghai, Kunming, Urumqi, Suzhou, and Changsha. In each region, we applied a stratified cluster random sampling method to select one to two junior high or high schools based on their geographical distribution and type. Within these schools, we randomly selected natural classes and then chose approximately 180 male and female students aged 12–17 from each age group, ensuring representation across different demographics. This process resulted in a total of 10,327 adolescents (5231 males and 5096 females) to participate in the study (**Figure 1**). The study was approved in advance by the Human Subject Protection Committee of East China Normal University (approval number: HR319-2021), and student participants and their parents provided informed consent.

### 2.2 Methods

#### 2.2.1 Sedentary behavior and screen time questionnaire

The sedentary behavior of participants was evaluated by administering the ‘Evaluation of physical activity level of children and adolescents aged 7–18 years old’ questionnaire (16), which asks participants about their activity levels over the past week. This includes questions on sedentary behavior items (video, traffic, social and other categories), days of participation, average duration of each activity, etc. This measure has been evaluated and tested by many professionals in the fields of children’s health, psychology, and sports. In order to assess the reliability of the questionnaire, we administered the survey to the same group of participants twice over a two-week period. The test-retest reliability coefficient was calculated to be 0.606, indicating a moderate degree of consistency in the participants’ responses between the two tests. This result is considered acceptable in social science research as it reflects the consistency and stability of the questionnaire in measuring sedentary behavior. The correlation between the sedentary behavior part of the questionnaire and the sedentary behavior duration measured by the three-axis accelerometer was 0.689, proving that it has good validity. Studies from various countries indicate that the average daily sedentary behavior duration for children and adolescents is about eight hours (9, 17, 18); therefore, this study used an average daily sedentary behavior duration of 8 hours as the cutoff point, with those participants spending ≥8 hours/day sitting considered the high sedentary behavior group, and those sitting <8 hours/day the low sedentary behavior group. An average daily screen time duration of two hours was used as the cutoff point, with those using screens for ≥2 hours/day categorized as the high screen time group and those with <2 hours/day of screen time as the low screen time group (16).

#### 2.2.2 Non-suicidal self-injury behavior assessment questionnaire

This study investigated participants’ engagement in NSSI behavior within the past year. This was evaluated using the measure “Adolescent NSSI Behavior Assessment Questionnaire” (19), which was compiled by Wan Yuhui ‘s team. The Cronbach’s α coefficient of the questionnaire was 0.921, the retest reliability was 0.843, and the correlation coefficient of criterion validity was 0.859, indicating that the questionnaire had high credibility and stability. It can be used as an evaluation tool to assess NSSI behavior in adolescents. According to the form and frequency of self-injury behavior, participants with a frequency of NSSI behavior of more than five times in the past year were categorized as the ‘frequent NSSI behavior group’, those engaging one-to-four times were designated the ‘occasional NSSI behavior group’, and those with zero occurrences of NSSI behavior formed the ‘no NSSI behavior group’.

### 2.3 Quality control

The team of investigators administering the surveys comprised teachers and graduate students who had passed the training and assessment. They were divided into different groups to visit schools in each province at the same time. Before administering the questionnaire, the investigators explained the purpose, significance, and requirements of the study to the participants using the guidance language prepared in advance. Any participant questions or issues were addressed by the investigators. Participants used an anonymous number to fill out the questionnaire. The questionnaire was distributed and recovered immediately after completion. When questionnaires were collected, investigators checked they had been filled in accurately, and participants were asked to complete any wrong or missing items in time to ensure the survey’s validity.

### 2.4 Statistical methods

SPSS 25.0 software (IBM, NY, USA) was utilized to conduct the statistical analyses. Missing data were addressed using multiple imputation methods to ensure the robustness of the research findings. Descriptive analyses were performed on the static behavior of adolescents and the basic characteristics of NSSI. Chi-square tests were performed to compare the detection rates of NSSI for different durations of sedentary behavior and screen time. For the binary logistic regression analysis to explore the impact of sedentary behavior on NSSI among adolescents. The presence or absence of NSSI was the dependent variable (yes = 1, no = 0), and the different durations of sedentary behavior and screen time were the independent variables. Potential confounding factors were controlled for, such as school stage, gender, family type, sleep compliance and study time (**Supplementary Tables S1–S3**), with daily sedentary behavior duration < 8 hours and daily screen time < 2 hours as the reference. *P*<0.05 was considered statistically significant.

## 3 Research results

### 3.1 Basic characteristics of adolescents’ sedentary behavior

**Figure 2:**
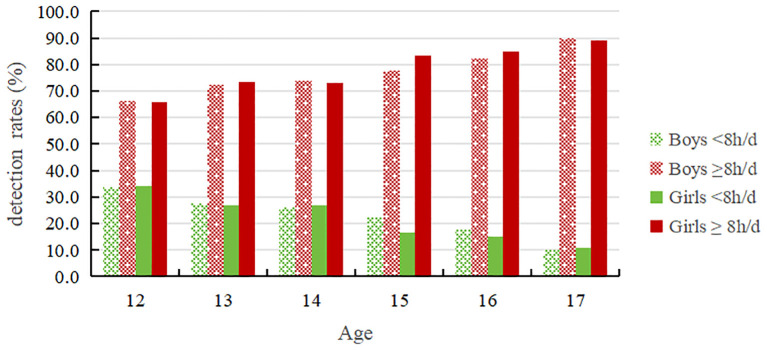
12-17 years old adolescents' detection rates in different duration groups of sedentary behaviors.

As seen in **Table 1** and **Figure 2**, the rate of adolescents’ daily sedentary behavior duration ≥8h shows an increasing trend with age, from 65.9% at the age of 12 to 89.4% at the age of 17. Additionally, there was a significant difference in the prevalence of daily sedentary behavior duration ≥8 hours among adolescents of different ages (*P* < 0.01). Sedentary behavior duration among boys increased from 66.1% at the age of 12 to 89.8% at the age of 17; in girls, it increased from 65.7% at the age of 12 to 89.0% at the age of 17. The overall detection rate of girls’ daily sedentary behavior duration ≥8 hours was 78.3%, higher than boys’ 77.0%.

**Table 1:** 12-17 years old adolescent’ detection rates in different duration groups of sedentary behaviors (%).

Age	N	Boys		N	Girls		N	Total	
		<8h/d	≥8h/d		<8h/d	≥ 8h/d		<8h/d	≥ 8h/d
12	880	33.9	66.1	826	34.3	65.7	1706	34.1	65.9
13	861	27.6	72.4	832	26.7	73.3	1693	27.2	72.8
14	866	26.2	73.8	859	26.9	73.1	1725	26.6	73.4
15	879	22.2	77.8	879	16.7	83.3	1758	19.5	80.5
16	891	17.7	82.3	855	15.1	84.9	1746	16.4	83.6
17	854	10.2	89.8	845	11.0	89.0	1699	10.6	89.4
*χ* ^2^	167.629			194.285			353.881		
*P* value	0.000			0.000			0.000		
Total	5231	23.0	77.0	5096	21.7	78.3	10327	22.3	77.7

**Figure 3:**
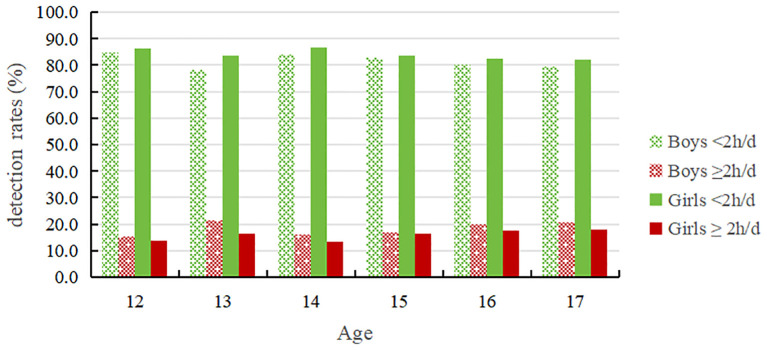
12-17 years old adolescents' detection rates in different duration groups of screen time.

**Table 2** and **Figure 3** demonstrates a fluctuating upward trend in the rate of adolescents’ daily screen time ≥2h with increasing age, with a rate of 14.7% at age 14 and then gradually increasing to 19.4% at age 17. Additionally, there was a significant difference in the prevalence of daily screen time ≥2 hours among adolescents of different ages (*P* < 0.01). The detection rate for boys’ daily screen time ≥2 hours was 18.4%, higher than the rate for girls (16.0%). The detection rate for boys’ daily screen time ≥2 hours was lowest at the age of 12(15.3%) and highest at the age of 17(20.7%); The percentage of girls with daily screen time use of ≥2 hours was lowest at the age of 12(13.8%) and highest at the age of 17, reaching 18.1%.

**Table 2:** 12-17 years old adolescent detection rates in different duration groups of screen time (%).

Age	N	Boys		N	Girls		N	Total	
		<2h/d	≥2h/d		<2h/d	≥ 2h/d		<2h/d	≥ 2h/d
12	880	84.7	15.3	826	86.2	13.8	1706	85.4	14.6
13	861	78.4	21.6	832	83.5	16.5	1693	80.9	19.1
14	866	83.8	16.2	859	86.7	13.3	1725	85.3	14.7
15	879	83.0	17.0	879	83.5	16.5	1758	83.3	16.7
16	891	80.2	19.8	855	82.5	17.5	1746	81.3	18.7
17	854	79.3	20.7	845	81.9	18.1	1699	80.6	19.4
*χ* ^2^	19.630			12.352			28.576		
*P* value	0.001			0.030			0.000		
Total	5231	81.6	18.4	5096	84.0	16.0	10327	82.8	17.2

### 3.2 The incidence and behavioral characteristics of non-suicidal self-injury among adolescents

**Table 3** shows that the total detection rate of NSSI behavior among adolescents over the past year was 25.1 %. The detection rates of occasional NSSI behavior and frequent NSSI behavior were 11.0 % and 14.1 %, respectively. The detection rate of NSSI behavior first increased with increasing age and then decreased. At the age of 14, the total detection rate for NSSI behavior was the highest at 27.9 %. At the age of 15, the detection rate of frequent NSSI behavior was the highest at 16.5 %. Moreover, there was a significant difference in the prevalence of NSSI behavior among adolescents of different ages (*P* < 0.01). Comparing the sexes, the total NSSI behavior detection rates for male and female students were 23.6 % and 26.7 %, respectively. Female students exhibited higher rates of NSSI behavior and occasional and frequent NSSI behavior than male students.

**Table 3:** Detection of NSSI in adolescents of different genders and ages(%).

Age	N	Boys			N	Girls			N	Total		
		No	Occasional	Frequently		No	Occasional	Frequently		No	Occasional	Frequently
12	880	77.6	10.8	11.6	826	73.5	10.2	16.3	1706	75.6	10.5	13.9
13	861	81.2	8.5	10.3	832	71.2	11.2	17.7	1693	76.3	9.8	13.9
14	866	74.7	11.8	13.5	859	69.6	11.8	18.6	1725	72.2	11.8	16.1
15	879	73.3	9.8	17.0	879	72.5	11.5	16.0	1758	72.9	10.6	16.5
16	891	74.4	12.2	13.4	855	76.8	10.8	12.4	1746	75.6	11.5	12.9
17	854	77.3	11.7	11.0	845	76.2	11.8	12.0	1699	76.8	11.8	11.5
χ^2^	32.614				26.833				31.147			
*P* value	0.000				0.003				0.001			
Total	5231	76.4	10.8	12.8	5096	73.3	11.2	15.5	10327	74.9	11.0	14.1

### 3.3 Comparison of non-suicidal self-injurious behavior in adolescents under different durations of sedentary behavior

**Figure 4:**
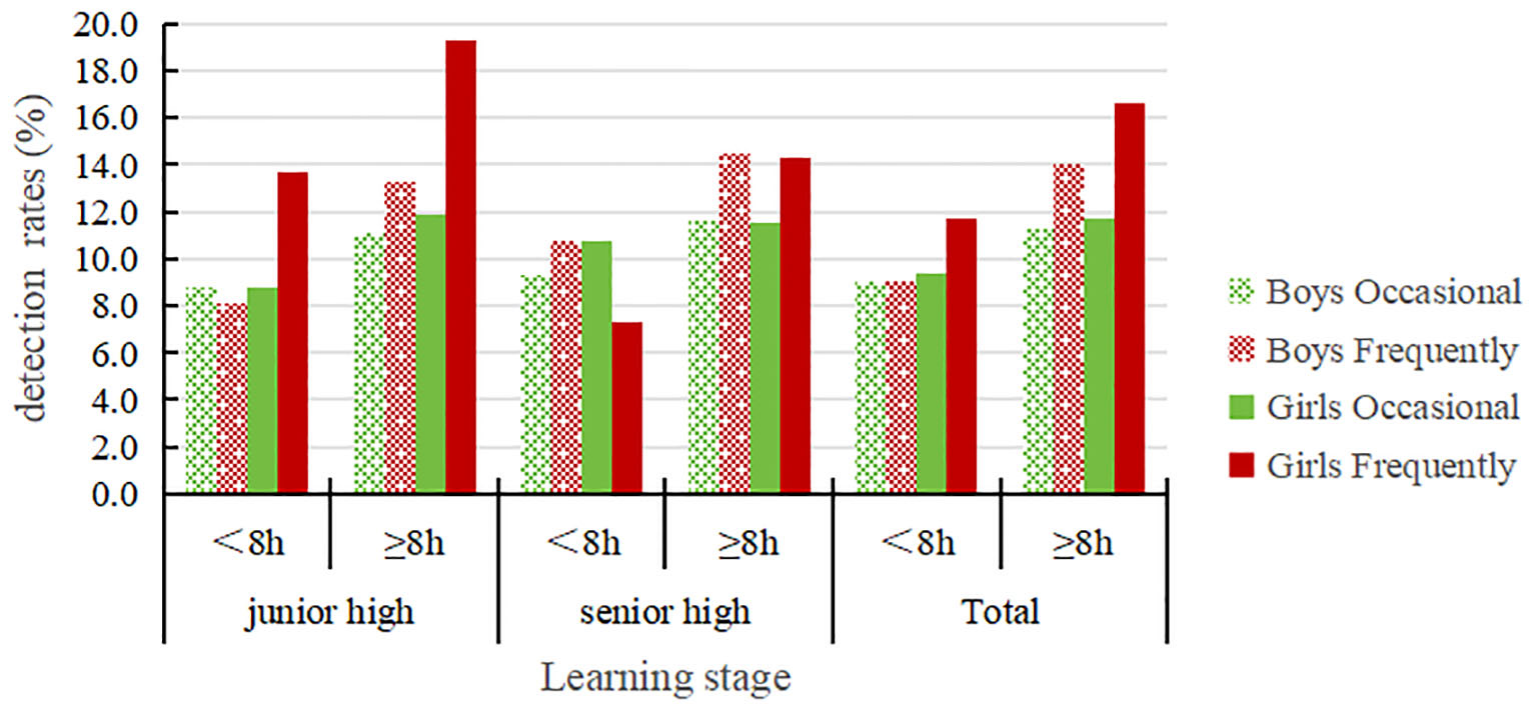
The detection of NSSI in adolescents with different sedentary behavior duration groups.

As can be seen from **Table 4** and **Figure 4**, the detection rates of occasional NSSI and frequent NSSI for junior and senior high school students with sedentary behavior durations ≥8h are both higher than those with sedentary behavior duration <8h and the differences are statistically significant (*P* < 0.01). For both male and female students, those with sedentary behavior durations ≥8h have higher detection rates for occasional and frequent NSSI than those with sedentary behavior durations <8h; the differences are statistically significant (*P* < 0.01). Additionally, in different sedentary behavior duration groups, the detection rates of occasional NSSI and frequent NSSI for female students were higher than those for male students.

**Table 4:** The detection of NSSI in adolescents with different sedentary behavior duration groups(%).

Learning stage	Time Division	Boys			*χ* ^2^	*P value*	Girls			*χ* ^2^	*P value*	Total			*χ* ^2^	*P value*
		N	Occasional	Frequently			N	Occasional	Frequently			N	Occasional	Frequently		
junior high	<8h	805	8.8	8.1	20.085	0.000	761	8.8	13.7	20.050	0.000	1566	8.8	10.8	^39.284^	^0.000^
	≥8h	1893	11.1	13.3			1794	11.9	19.3			3687	11.5	16.2		
senior high	<8h	398	9.3	10.8	6.476	0.039	344	10.8	7.3	13.687	0.001	742	10.0	9.2	^18.168^	^0.000^
	≥8h	2135	11.6	14.5			2197	11.5	14.3			4332	11.5	14.4		
Total	<8h	1203	9.0	9.0	29.246	0.000	1105	9.4	11.7	23.470	0.000	2308	9.2	10.3	^53.198^	^0.000^
	≥8h	4028	11.3	14.0			3991	11.7	16.6			8019	11.5	15.3		

**Figure 5:**
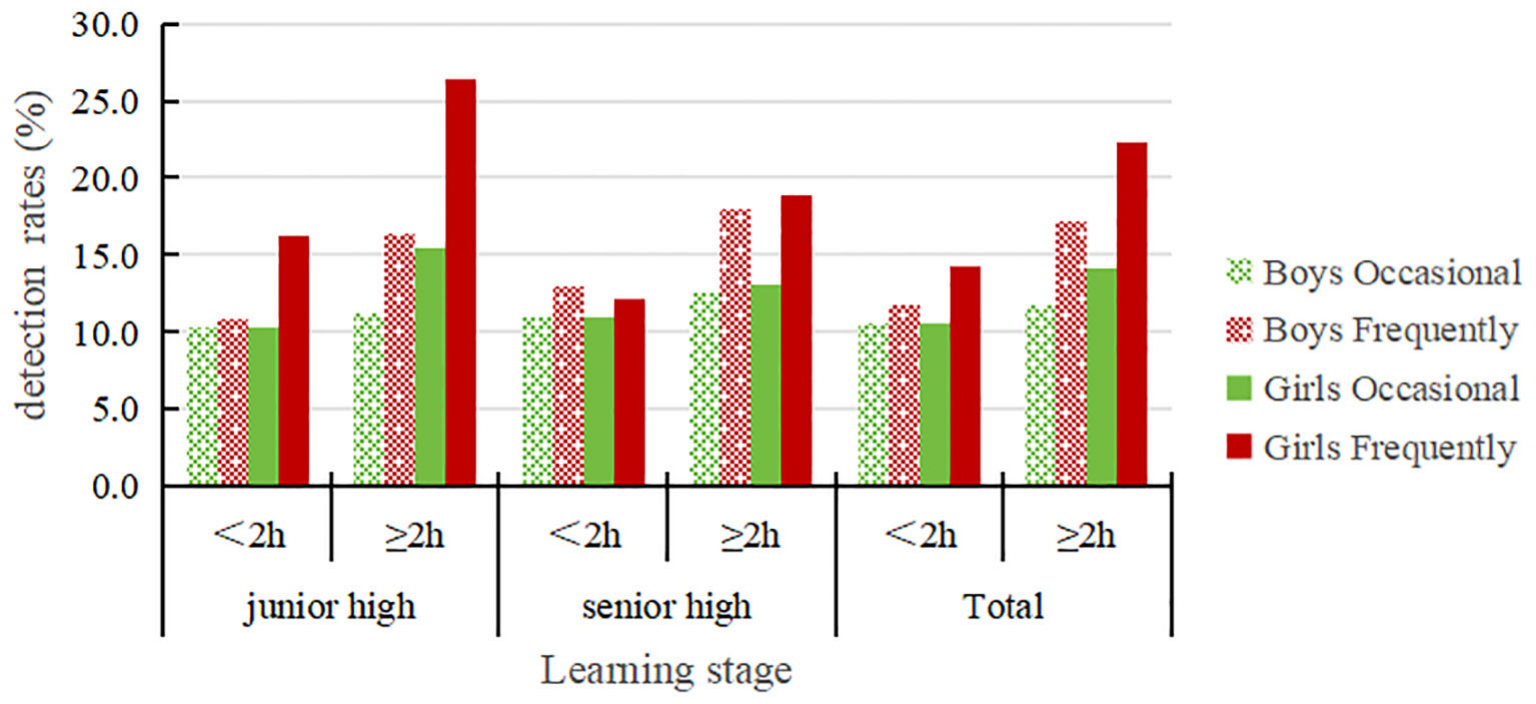
The detection of NSSI in adolescents with different screen time groups.

**Table 5** and **Figure 5** shows that the detection rates for both occasional and frequent NSSI in junior and senior high school students with screen time ≥2h are higher than those with screen time <2h and the differences are statistically significant (*P* < 0.01). For both male and female students with screen time ≥2h, the detection rates of occasional and frequent NSSI are both higher than for students with screen time <2h; the differences are statistically significant (*P* < 0.01). Additionally, female students in the different screen time groups had higher rates of, occasional and frequent NSSI than male students.

**Table 5:** The detection of NSSI in adolescents with different screen time groups(%).

Learning stage	Time Division	Boys			*χ^2^*	*P value*	Girls			*χ^2^*	*P value*	Total			*χ^2^*	*P valu*e
		N	Occasional	Frequently			N	Occasional	Frequently			N	Occasional	Frequently		
Junior high	<2h	2223	10.3	10.8	13.185	0.001	2191	10.3	16.2	36.204	0.000	4414	10.3	13.4	40.169	0.000
	≥2h	475	11.2	16.4			364	15.4	26.4			839	13.0	20.7		
senior high	<2h	2045	10.9	13.0	10.515	0.005	2092	11.0	-12.2	17.823	0.000	4137	11.0	12.6	27.776	0.000
	≥2h	488	12.5	18.0			449	13.1	18.9			937	12.8	18.5		
Total	<2h	4268	10.6	11.8	23.873	0.000	4283	10.6	14.2	48.406	0.000	8551	10.6	13	66.487	0.000
	≥2h	963	11.8	17.2			813	14.1	22.3			1776	12.9	19.5		

### 3.4 Logistic regression analysis of sedentary behavior and non-suicidal self-injurious behavior

**Table 6** shows that in Model 1, no variables were adjusted, and in Model 2, after controlling for factors such as school section, gender, family structure, sleep standard achievement, and study time, the NSSI detection rate for the group with daily sedentary behavior duration ≥8 hours was 1.393times that of the group with <8 hours (*β*= 0.332, *P* < 0.001). The NSSI detection rate for the group with daily screen time ≥2 hours was 1.569 times that of the group with <2 hours (*β*= 0.451, *P* < 0.01).

**Table 6:** Association of sedentary behavior duration and screen time with NSSI in adolescents.

Variable		Model 1					Model 2				
		*β*	Std. Error	*ORvalue*	95%*CI*	*Pvalue*	*β*	Std. Error	*ORvalue*	95%*CI*	*Pvalue*
Average daily SB	<8h			1					1		
	≥8h	0.41	0.059	1.506	1.342∼1.690	<0.001	0.332	0.064	1.393	1.229∼1.580	<0.001
Average daily screen time	<2h			1							
	≥2h	0.432	0.057	1.541	1.378∼1.723	<0.001	0.451	0.058	1.569	1.401∼1.758	<0.001

Model 1 did not adjust the variables. Model 2 adjusted the factors of school stage, gender, family type, sleep compliance, and study time.

## 4 Discussion

This study found that the duration of sedentary behavior and screen time showed an increasing trend with increasing age. Both domestic and foreign studies have shown that adolescents’ sedentary behavior duration is between 8 and 9.5 hours per day (11, 12, 17, 20). Our results are similar, indicating that the sedentary behavior duration of adolescents has not improved in recent years. In addition, our study also found a positive correlation between male sex, age/grade, and sedentary behavior; that is, the sedentary behavior duration of male students was longer than that of female students, and the sedentary behavior duration of senior students was longer than that of junior students (21). The duration of sedentary behavior is especially long in senior high school, which may be related to the curriculum setting in senior high school, including the number of class hours, the length of evening self-study, and the pressure of further education and schoolwork. In addition, studies have shown that with increasing age, adolescents tend to choose sedentary activities, and girls grow in this area faster than boys, which may be due to differences in the physical and mental development of the sexes (22).

Previous studies report that the screen time of children and adolescents in China is slightly higher than that found by this study (22). However, the present research did not differentiate between weekends and weekdays, and many studies have shown that teens spend significantly more time using screens on the weekend than midweek (23, 24). In general, this study shows that the average daily screen time of adolescents in China has declined and is lower than that of some developed countries. In recent years, China has attached great importance to the problems of increasing rates of myopia and a reduction In physical activity in children and adolescents and has taken corresponding measures to intervene; this may be one of the reasons for the decline of screen time in adolescents. However, with the popularity of various electronic devices, teenagers may increase their use of them, so it is necessary to continue to pay attention to and manage the sedentary behavior and screen time within a reasonable range.

Our study found that the total detection rate of NSSI behavior among adolescents in Chinese four cities was 25.1 %, which was slightly lower than the results of a domestic meta-analysis (28.2 %) (25), but similar to the detection rate of developed countries such as Italy and the Netherlands (26, 27). These findings suggest that while there are global trends in NSSI behavior, the cultural context of China, with its unique social and familial expectations, may influence the expression and reporting of NSSI behaviors. The lower detection rate compared to the meta-analysis might reflect cultural stigma or differences in behavioral reporting within Chinese adolescents. We also found that the incidence of NSSI behaviors among adolescents varies significantly with age, with 12–15 years being the high-incidence age group. Previous research indicates that the detection rate of NSSI behaviors in adolescence shows an inverted trend, peaking at 15 years of age before beginning to decline (28). In this study, the detection rate peaked at ages 14–15 and was significantly higher than in the other age groups. Overall, the NSSI behavior of adolescents showed a trend of first increasing and then decreasing with age, which is similar to the results of the aforementioned studies. This may be related to the fact that adolescents in the 14–15 age group are more likely to experience negative psychological emotions due to facing sensitive issues such as academic pressure, sexual maturity, and the rebellious period (29). Combined with the traits of irritability and impulsivity common in adolescents, this age group is more likely to resort to self-injurious behavior as a means to vent negative emotions. Preventing and intervening in NSSI behavior among adolescents during this high-incidence period will be crucial in reducing the current high occurrence of NSSI behavior.

In addition, we found that the NSSI behavior detection rate in girls was significantly higher than that in boys, consistent with previous research results (3, 30). However, sex differences in NSSI behavior among adolescents have not yet been established, and results are conflicting. Some studies have shown that the detection rate of NSSI behavior in boys is higher than that in girls (31), while others have pointed out that there is no significant difference between the sexes (32). These contradictory results may be related to the various studies’ evaluation methods and the age characteristics of the participants. The results of our study show that NSSI behavior is more common among girls in early adolescence, while in later adolescence, boys are more likely to engage in such behavior. This suggests that age-characteristic differences between participants in different studies may be a reason for the sex differences in the incidence of NSSI among adolescents. In terms of the types of self-injurious behavior exhibited by boys and girls, Hu Ya’s study (33) shows that boys are more inclined to engage in behaviors such as hitting, striking hard objects, and injuring themselves, while girls tend to adopt behaviors such as pinching, scratching, biting, piercing, and abrasions. The sex differences found in NSSI behavior among adolescents may also be related to the differences in the physical and mental development of boys and girls. Some studies have pointed out that NSSI behavior in girls is closely related to attempted suicide, while boys are more likely to engage in NSSI behavior to externalize their inner emotions (34). These results suggest that subsequent research should further refine their sample based on different sexes and ages, to conduct relevant explorations targeting different high-risk groups with distinct characteristics. This will provide a reference and a basis for developing more targeted prevention and control measures and intervention programs.

This study also found that longer sedentary behavior duration and screen time significantly increased the risk of NSSI behavior in adolescents. Other research suggests potential physiological and psychological mechanisms, such as the impact of sedentary behavior on mood regulation and the psychological effects of increased isolation and depressive symptoms, which are associated with NSSI (35). Foreign studies have found that health risk behavior is related to an increase in the detection rate of self-injury behavior and self-injury intention, including longer leisure-time sedentary behavior, which is consistent with the results of this study (36). However, there are few studies on the relationship between sedentary behavior and adolescent NSSI behavior at home and abroad. The present stud’s results indicate that even after controlling for related factors, a positive correlation between the total duration of sedentary behavior and adolescent NSSI behavior remains. Considering demographic factors, family structure, nutritional status, and sleep patterns, the total duration of sedentary behavior still has a significant relationship with adolescent NSSI behavior. This suggests that sedentary behavior may have an independent negative effect on adolescent NSSI behavior, but it may also be influenced by other related factors. Therefore, further research is needed to strengthen the understanding of the relationship between sedentary behavior and adolescent NSSI behavior.

In addition, several studies have found that screen time has an impact on the risk of self-injury behavior in adolescents, and too much screen time is associated with a high NSSI detection rate (1). This is consistent with the findings of this study, which show that spending more than two hours per day in front of a screen significantly increases the risk of NSSI behavior among adolescents. Watching videos related to self-injury behavior may induce children and adolescents to follow suit, and screen time can also lead to insufficient sleep and various related adverse psychological symptoms, increasing the risk of NSSI behavior (37). Overseas studies (38) have found that online chatting and sending instant messages are associated with NSSI behaviors among children and adolescents. Similarly, Liu et al.’s study of middle school students in 10 cities in China (39) found that excessive screen time is related to a higher NSSI detection rate. Hawto’s research also found that excessive screen time not only has a serious negative impact on the mental health of adolescents (1) but is also a risk factor for repeated self-injury and suicide. The present study also found that the detection rate of self-injurious behavior among adolescents engaging in excessive screen use is 10% higher than that of those with less than 2 hours of screen time per day. Research has found that up to 90% of videos with high viewership on YouTube contain non-character videos engaging in self-injurious behavior, and 28% of character-based videos also contain this type of behavior (37). Watching videos related to self-injurious behavior may lead children and adolescents to imitate, which is one of the reasons for the increased risk of NSSI associated with excessive screen time. Excessive screen time will also cause sleep deprivation and the emergence of various poor psychological symptoms such as depression and anxiety, which will further increase the risk of NSSI. Internet addiction will greatly increase the screen time of middle school students, and studies have shown that there is a close relationship between NSSI behavior and internet addiction among middle school students (40, 41). Internet addiction and suspected internet addiction are both risk factors for NSSI behavior among middle school students, suggesting that NSSI and internet addiction behavior may have a common development pattern and interact with one another. The internet provides an information source about adolescent NSSI behavior, and students who engage in NSSI behavior use the internet to alleviate negative emotions. Adolescents have a strong curiosity and desire to imitate new things, cannot view things scientifically, and are prone to psychological and behavioral problems. If the internet provides improper guidance, it will lead to a high incidence of NSSI among adolescents, resulting in a significant positive correlation between screen time and NSSI behavior.

While this study reveals a significant positive correlation between adolescent's sedentary behavior, particularly screen time, and NSSI, it is not without limitations. The cross-sectional nature of our data limits our ability to infer causality, and the reliance on self-reported measures of sedentary behavior and NSSI may introduce reporting biases. Our results may have broad generalizability; however, we acknowledge the need for further validation of these findings in different cultural, socioeconomic, and geographical contexts to enhance the external validity of our conclusions.

Recognizing the limitations of our study, future research should incorporate a wider range of potential confounding factors, including mental health status, social support networks, and family environment, to better understand the relationship between sedentary behavior and NSSI. A multicenter, multi-population approach would also strengthen the assessment of the generalizability and applicability of intervention measures, addressing the limitations of our single-setting study.

Despite the limitations, our findings support the development of clinical interventions targeting sedentary behavior to prevent NSSI. Future research should evaluate the effectiveness of interventions such as school-based physical activity promotion, parent education, and digital health applications in reducing screen time and promoting physical activity among adolescents. It is crucial to explore the applicability of these interventions across different populations and settings to maximize their impact.

In summary, this study provides a new perspective on the prevention of NSSI behavior among adolescents and points the way for future intervention research and clinical practice.

## 5 Conclusions

The results of this study confirm that sedentary behavior, especially screen time, is closely related to the occurrence of non-suicidal self-injury behavior in adolescents, indicating that it is feasible to intervene in and prevent NSSI behavior in adolescents by encouraging physical activity.

Based on the study’s findings, we propose the following specific recommendations:

Schools and families should restrict adolescent’ sedentary behavior and screen time, encouraging more physical activity.

Public health policies should promote physical activities among adolescents to prevent NSSI behavior.

Future research should further explore interventions to reduce sedentary behavior and their effectiveness in preventing NSSI behavior.

Through these measures, we can anticipate significant progress in reducing NSSI among adolescents, creating a more positive and healthier environment for their growth.

## Data Availability

The original contributions presented in the study are included in the article/**Supplementary Material**. Further inquiries can be directed to the corresponding author/s.
