# Supplementary material for: The relationship between sedentary behavior and non-suicidal self-injury behavior among adolescents in China
Source: Front Psychiatry. 2024 Dec 4;15:1489707. doi: 10.3389/fpsyt.2024.1489707 (PMC11652529; doi:10.3389/fpsyt.2024.1489707)
Supplement: Supplementary file 1 [file Table1.docx]

Supplementary Material

# Supplementary Tables

| **Table S1** The detection of NSSI in adolescents with different family type（%） | | | | | |
| --- | --- | --- | --- | --- | --- |
| Family type | N | Occasional | Frequently | χ^2^ | *P value* |
| Nuclear family | 7032 | 11.0 | 13.3 | 39.029 | 0.000 |
| Stem family | 2340 | 10.8 | 14.3 |  |  |
| Joint family | 125 | 7.2 | 21.6 |  |  |
| Single-parent family | 527 | 10.4 | 18.4 |  |  |
| Blended family | 303 | 14.5 | 21.1 |  |  |
| Total | 10327 | 11.0 | 14.1 |  |  |

| **Table S2** The detection of NSSI in adolescents with different sleep compliance grouping（%） | | | | | |
| --- | --- | --- | --- | --- | --- |
| Sleep compliance grouping | N | Occasional | Frequently | χ^2^ | *P value* |
| None Meet Standards | 2498 | 12.8 | 18.6 | 130.040 | 0.000 |
| School Days Not Meet, Weekends Meet | 6018 | 11.3 | 13.8 |  |  |
| School Days Meet, Weekends Not Meet | 290 | 7.6 | 10.7 |  |  |
| Both Meet Standards | 1521 | 7.4 | 8.7 |  |  |
| Total | 10327 | 11.0 | 14.1 |  |  |

| **Table S3** Correlation between Study Time and Total NSSI Frequency(r) | | | |
| --- | --- | --- | --- |
| Age | Boys | Girls | Total |
| 12 | 0.040 | -0.022 | 0.011 |
| 13 | -0.009 | 0.064 | 0.035 |
| 14 | 0.510 | 0.056 | 0.055* |
| 15 | 0.078* | 0.029 | 0.055* |
| 16 | 0.058 | 0.125** | 0.091** |
| 17 | 0.092** | 0.000 | 0.045 |
| Total | 0.059** | 0.029* | 0.044* |
| Note: * indicates P<0.5, ** indicates P<0.1. | | | |
